# Trimethoprim-sulfamethoxazole prophylaxis during treatment of granulomatosis with polyangiitis with rituximab in the United States of America: a retrospective cohort study

**DOI:** 10.1186/s13075-023-03114-7

**Published:** 2023-07-29

**Authors:** Arielle Mendel, Hassan Behlouli, Cristiano Soares de Moura, Évelyne Vinet, Jeffrey R. Curtis, Sasha Bernatsky

**Affiliations:** 1 Division of Rheumatology, McGill University Health Centre, 1560 Cedar Avenue, Montreal, QC H3G 1A4 Canada; 2 Centre for Outcomes Research and Evaluation, Research Institute of the McGill University Health Centre, 5252 de Maisonneuve Blvd. W., Montreal, QC H4A 3S9 Canada; 3 Division of Clinical Immunology & Rheumatology, University of Alabama at Birmingham, 2000 6th Avenue South, Birmingham, AL 35255 USA

**Keywords:** ANCA-associated vasculitis, Granulomatosis with polyangiitis, Rituximab, Cohort studies, Antibiotic prophylaxis, Trimethoprim-sulfamethoxazole

## Abstract

**Background:**

Antibiotic prophylaxis is recommended during ANCA-associated vasculitis (AAV) induction. We aimed to describe the frequency, persistence, and factors associated with trimethoprim-sulfamethoxazole (TMP-SMX) use in an adult population sample with granulomatosis with polyangiitis (GPA) treated with rituximab (RTX).

**Methods:**

We identified adults with GPA treated with RTX within the Merative™ Marketscan® Research Databases (2011–2020). TMP-SMX prophylaxis was defined as a $\ge$ 28-day prescription dispensed within a month of starting RTX. We estimated TMP-SMX persistence, allowing prescription refill gaps of 30 days. Multivariable logistic regression and Cox proportional hazards regression assessed the factors associated with baseline TMP-SMX use and persistence, respectively. Covariates included age, sex, calendar year, insurance type, immunosuppressant use, hospitalization, and co-morbidities.

**Results:**

Among 1877 RTX-treated GPA patients, the mean age was 50.9, and 54% were female. A minority (*n* = 426, 23%) received TMP-SMX with a median persistence of 141 (IQR 83–248) days. In multivariable analyses, prophylaxis was associated with prednisone use in the month prior to RTX ($\ge$ 20 mg/day vs none, OR 3.96; 95% CI 3.0–5.2; 1–19 mg/day vs none, OR 2.63; 95% CI 1.8–3.8), and methotrexate use (OR 1.48, 95% CI 1.04–2.1), intensive care (OR 1.95; 95% CI 1.4–2.7), and non-intensive care hospitalization (OR 1.56; 95% CI 1.2–2.1) in the 6 months prior to RTX. Female sex (OR 0.63; 95% CI 0.5–0.8) was negatively associated with TMP-SMX use.

**Conclusions:**

TMP-SMX was dispensed to a minority of RTX-treated GPA patients, more often to those on glucocorticoids and with recent hospitalization. Further research is needed to determine the optimal use and duration of TMP-SMX prophylaxis following RTX in AAV.

**Supplementary Information:**

The online version contains supplementary material available at 10.1186/s13075-023-03114-7.

## Introduction

The anti-neutrophil cytoplasm antibody (ANCA)-associated vasculitides (AAV) are life-threatening systemic necrotizing small vessel vasculitides [1]. Rituximab (RTX) has increasingly become a first-line induction and maintenance treatment of severe granulomatosis with polyangiitis (GPA) and microscopic polyangiitis (MPA) [2, 3]. However, serious infections, which occur in approximately one-quarter of patients during AAV treatment [4–10], are a significant complication and may result in death [11, 12]. Strategies to reduce serious infections are therefore a priority.

Low-dose trimethoprim-sulfamethoxazole (TMP-SMX) prophylaxis is recommended during AAV induction to prevent *Pneumocystis jirovecii* pneumonia (PJP) [13–16]. Furthermore, an early randomized controlled trial [17] and recent observational studies [18, 19], including a post hoc analysis of the RAVE trial [20], found an association between TMP-SMX use and reduced overall (all-cause) serious infections in AAV. Recently, the American College of Rheumatology [3] and the Canadian Vasculitis Research Network [2] recommended TMP-SMX prophylaxis (or alternatives, in the case of allergy/intolerance) during RTX induction and for at least 6 months following the last RTX dose. The British Society of Rheumatology suggests continuing prophylaxis during RTX maintenance therapy, especially in high-risk patients, such as those with structural lung disease, prolonged glucocorticoid use, and increased age [21].

In the decade leading up to these recommendations, real-world patterns of TMP-SMX prophylaxis in AAV following treatment with RTX are unknown. Our objective was to describe the frequency and persistence of TMP-SMX prophylaxis in patients with granulomatosis with polyangiitis (GPA) treated with RTX in a US population sample and determine factors associated with prophylaxis.

## Methods

### Data source

We identified patients with GPA within the Merative™ Marketscan® Research Databases, which comprises US administrative health data for beneficiaries of employer-sponsored health insurance and some smaller commercial insurance plans (patients aged < 65), Medicare-eligible retirees with employer-sponsored Medicare supplemental plans (patients aged ≥ 65), and a subsample of Medicaid enrollees from participating state Medicaid programs. In these data, International Classification of Diseases (ICD) diagnostic codes (from billing and hospitalizations), Current Procedural Terminology (CPT) codes, and National Drug Codes (NDC) identify medical claims from physician outpatient visits, hospitalizations, procedures, and prescription drug dispensations. Prior studies have evaluated GPA epidemiology and cost within MarketScan [10, 22, 23].

### Ethical approval

This study complies with the Declaration of Helsinki and was approved by the McGill University Faculty of Medicine Institutional Review Board (22-01-037).

### Cohort selection

**Fig. 1:**
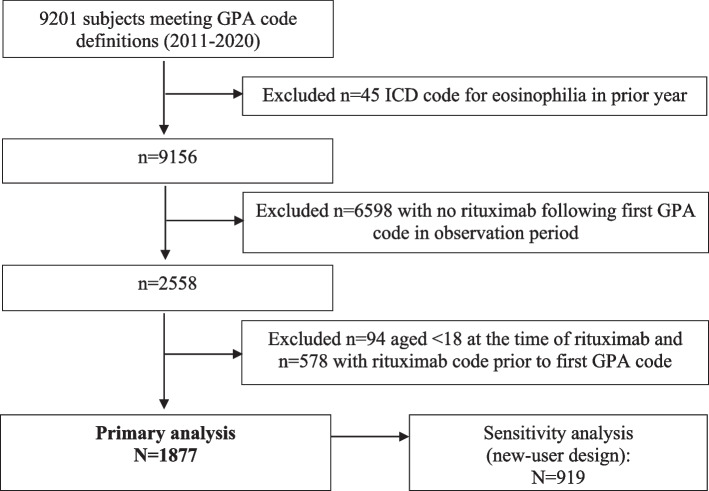
Cohort assembly. *Abbreviations*: GPA, granulomatosis with polyangiitis; ICD, International Classification of Diseases

We identified patients with GPA insured between January 1, 2011, and December 31, 2020, according to previously established case definitions [10]. Subjects were required to have at least 1 inpatient claim or 2 outpatient claims at least 30 days apart (but not more than 12 months apart) with a diagnostic code for GPA (i.e., ICD-9 446.4, ICD-10 M31.31, or M31.30) and at least one CPT or NDC code for RTX any time following the first GPA diagnostic code. The combination of ICD coding and specific medication use has a reported sensitivity of up to 94% and a positive predictive value of up to 79% for GPA [24]. We excluded subjects who were aged < 18 at the time of first (index) RTX, if they had any code for RTX prior to the first GPA diagnostic code, or if they had any diagnosis code for eosinophilia (ICD-9 228.3, ICD-10 D72.1) in the year prior to the first GPA code (in order to exclude subjects with eosinophilic granulomatosis with polyangiitis). We did not include MPA, as there is no specific ICD-9 code for this condition. As a sensitivity analysis, we restricted the study population to subjects with continuous eligibility in the database (for medical or drug benefits) for at least 6 months prior to the index RTX procedure code (new-user design, Fig. 1).

### Cohort characteristics and exposure definitions

Age, sex, insurance type (commercial, Medicare, Medicaid), and calendar year were determined at the time of index RTX. Because RTX doses are not available in the database, we defined the index RTX treatment as “induction” if it was followed by one or more additional RTX infusions in the following 30 days (to capture regimens of 375 mg/m^2^ weekly for 4 weeks or 1000 mg every 2 weeks for 2 doses [25]), and defined the index RTX as a “maintenance” treatment if there were no additional infusions in the following 30 days. As patients often start treatment with high-dose glucocorticoids in the weeks prior to receiving RTX induction, we captured prednisone dispensed in the 30 days prior to the index date and categorized the daily dose of the prescription as ≥ 20 mg/day, 1–19 mg/day, or non-use. A 6-month lookback period prior to index RTX also captured recent immunosuppressant use (oral or IV cyclophosphamide, methotrexate, azathioprine), physician visits, hospital admissions (with/without intensive care unit, ICU admission), and serious infections, defined as a hospitalization with a primary diagnosis code for a bacterial or unspecified infection (see Additional file 1: Table S1 for infection ICD codes). We also evaluated patient characteristics using ICD and/or procedure codes for the following disease features and/or co-morbidities anytime prior to index RTX: sinusitis, obstructive lung disease (includes chronic obstructive pulmonary disease, bronchiectasis, and asthma), interstitial lung disease, diabetes, glomerulonephritis, chronic kidney disease, and dialysis.

To identify individuals with potential TMP-SMX allergies or intolerance, we identified ≥ 28-day prescriptions for dapsone and atovaquone (PJP prophylaxis alternatives to TMP-SMX) dispensed in the 6 months prior to and following index RTX.

### Outcomes

The primary outcome was baseline TMP-SMX prophylaxis, defined as a ≥ 28-day prescription for TMP-SMX (to exclude intermittent antibiotic prescriptions for acute infection [26]), either dispensed prior to RTX with enough supply to overlap this RTX treatment or dispensed within the 30 days following the index RTX [27].

### Analysis

Baseline cohort characteristics were described at the time of index RTX treatment in the observation period, overall and stratified according to TMP-SMX user status. Continuous data were expressed as means (with standard deviation (SD)) or medians (with interquartile range (IQR)). Categorical data were summarized as counts and percentages. We assessed whether TMP-SMX use increased across the calendar year using the Cochran Armitage Trend test.

Univariable logistic regression analyses assessed the association of the following with baseline TMP-SMX use: age, sex, insurance type (commercial/Medicare vs Medicaid), calendar year period (2016–2020 vs 2011–2015), RTX induction (vs maintenance), hospital admission with and without ICU stay (each vs no hospitalization), having at least 1 co-morbid condition (prior ICD codes for lung disease as defined above, diabetes, chronic kidney disease, dialysis), prednisone use in the prior month (≥ 20 mg/day and 1–19 mg/day, each vs none), and use of other immunosuppressants in the prior 6 months. We hypothesized that mean prednisone ≥ 20 mg/day in the month preceding index RTX would be associated with TMP-SMX use, as this is the dose above which experts generally recommend PJP prophylaxis [28]. We expected that TMP-SMX use would be more frequent with RTX induction, as this is a higher risk period for infection. We developed a multivariable model that included relevant potential confounders.

In a pre-specified sensitivity analysis, we restricted the study population to those with 6 months of continuous insurance enrollment prior to index RTX (new user design), in order to limit the inclusion of prevalent RTX users. In the second sensitivity analyses, we liberalized the time window for starting prophylaxis to also include ≥ 28-day prescriptions for TMP-SMX dispensed any time in the 6 months following index RTX (as patients may delay filling prescriptions for a variety of reasons and start prophylaxis later than intended), within the whole study population and in the subgroup with continuous insurance enrollment for the 6 months post-RTX.

### Treatment persistence

In the subset of TMP-SMX users with 6 months of continuous insurance enrollment following RTX, we assessed the median duration (in days) of continuous TMP-SMX use (i.e., persistence) from the time of RTX, allowing a maximum refill gap of 30 days between consecutive prescriptions. Kaplan-Meier estimates determined the proportion of subjects remaining on TMP-SMX at 6 months. Cox proportional hazards regression assessed the association between baseline characteristics and time to TMP-SMX discontinuation. Subjects were censored if > 30 days passed with no new prescription dispensed, loss of insurance enrollment, or December 31, 2020, whichever came first. All analyses were performed in SAS (Version 9.4 TS Level 1M6).

## Results

### Study population

Of 9201 adults meeting GPA ICD code definitions (January 1, 2011, to December 31, 2020), 1877 (20%) received RTX following the first GPA code and met other cohort inclusion criteria (Fig. 1). About half of the cohort was female, and the mean age was 50.9 years at the time of the first (index) RTX infusion (Table 1). The majority (70%) of RTX was induction (according to the study definition). Most (74%) had commercial insurance, while 253 (13%) were covered under Medicare Advantage and 231 (12%) under Medicaid. In the 6 months prior to RTX, 774 (41%) had at least one hospital admission, 317 (17%) required ICU, and 172 (9%) had received dialysis. In the 30 days leading up to RTX, 850 (45%) used prednisone, including 630 (34%) who were dispensed prednisone ≥ 20 mg daily.

**Table 1:** Cohort characteristics, overall and according to trimethoprim-sulfamethoxazole (TMP-SMX) use

Characteristic at the time of first rituximab treatment	Overall cohort, *N* = 1877	TMP-SMX, *N* = 426	No TMP-SMX, *N* = 1451
Age, mean (SD)	50.9 (16)	48.4 (15)	51.6 (16)
Female sex (%)	1008 (54)	188 (44)	820 (57)
Insurance type, *n* (%)			
Commercial	1393 (74)	345 (81)	1048 (72)
Medicare	253 (14)	37 (9)	216 (15)
Medicaid	231 (12)	44 (10)	187 (13)
Year of index date, *n* (%)			
2011–2015	898 (48)	185 (43)	713 (49)
2016–2019	979 (52)	241 (57)	738 (51)
Rituximab treatment type, *n* (%)			
Induction	1314 (70)	323 (76)	991 (68)
Maintenance	563 (30)	103 (24)	460 (32)
Healthcare use in the prior 6 months			
Number of physician visits, mean (SD)	16.1 (15)	18.5 (14)	15.4 (14)
≥ 20 physician visits	577 (31)	164 (39)	413 (28)
Hospital admission, *n* (%)	774 (41)	236 (55)	538 (37)
Intensive care unit admission, *n* (%)	317 (17)	102 (24)	215 (15)
Prior serious infection	159 (9)	48 (11)	111 (8)
Disease features and/or co-morbidities, *n* (%)			
Sinusitis	491 (26)	116 (27)	371 (26)
Obstructive lung disease	393 (21)	92 (23)	297 (21)
Interstitial lung disease	90 (5)	19 (4)	71 (5)
Glomerulonephritis	208 (11)	46 (11)	162 (11)
Chronic kidney disease	435 (23)	91 (21)	344 (24)
Dialysis	172 (9)	40 (9)	132 (9)
Diabetes	273 (15)	56 (13)	217 (15)
Medication use, *n* (%)			
Prednisone 1–19 mg/day^a^	220 (12)	60 (14)	160 (11)
Prednisone ≥ 20 mg/day^a^	630 (34)	247 (58)	383 (26)
Cyclophosphamide^b^	61 (3)	14 (3)	47 (3)
Azathioprine^b^	92 (5)	20 (5)	72 (5)
Methotrexate^b^	202 (11)	61 (14)	141 (10)
Atovaquone^b^	43 (2)	4 (1)	39 (3)
Dapsone^b^	38 (2)	3 (1)	35 (2)

^a^Dispensed in the month prior to rituximab

^b^At least one prescription dispensed in the 6 months prior to rituximab

### TMP-SMX prophylaxis

Baseline TMP-SMX was dispensed to 426 (23%), with the majority (*n* = 314, 73% of TMP-SMX users) starting prescriptions prior to index RTX and continuing afterwards, and the remainder (*n* = 112) starting within the month following RTX. TMP-SMX was dispensed to 323/1314 (25%) induction recipients and 247/630 (39%) who were dispensed prednisone ≥ 20 mg daily in the previous month.

### Factors associated with baseline TMP-SMX use

In univariable analyses, baseline TMP-SMX use was associated with prednisone ≥ 20 mg/day (vs 0 mg, OR 4.92; 95% CI 3.84–6.33) and prednisone 1–19 mg/day (vs 0 mg, OR 2.86; 95% CI 2.00–4.06) in the month prior to RTX. Induction RTX (vs maintenance), hospitalization with and without ICU stay, prior use of methotrexate, and having a serious infection in the 6 months before RTX were also significantly associated with TMP-SMX use. Although TMP-SMX was more often dispensed in the 2016–2020 period (vs 2011–2015, OR 1.26; 95% CI 1.01–1.57), the *p* value for the calendar year trend was 0.06. Age (in years, OR 0.99; 95% CI 0.98–0.99) and female sex (OR 0.61; 95% CI 0.49–0.76) were negatively associated with prophylaxis. We did not find any clear associations between co-morbidities (lung disease, chronic kidney disease/dialysis, or diabetes) and TMP-SMX use.

In multivariable analyses, female sex (OR 0.63; 95% CI 0.50–0.80) and age in years (OR 0.99; 95% CI 0.98–0.99) remained negatively associated with TMP-SMX, while prednisone ≥ 20 mg/day (vs 0 mg, OR 3.96; 95% CI 3.04–5.19), prednisone 1–19 mg/day (vs 0 mg, OR 2.63; 95% CI 1.83–3.77), hospitalization with ICU (OR 1.95; 95% CI 1.39–2.73) and without ICU (OR 1.56; 95% CI 1.16–2.09), and methotrexate use (OR 1.48; 95% CI 1.04–2.09) remained associated with TMP-SMX (Table 2).

**Table 2:** Univariable and multivariable logistic regression analysis of factors associated with TMP-SMX use (*N* = 1877)

Characteristic at the time of first rituximab treatment	Univariable		Multivariable	
	OR	95% CI	OR	95% CI
Age (years)	0.99	0.98–0.99	0.99	0.98–0.99
Female sex	0.61	0.49–0.76	0.63	0.50–0.80
Rituximab induction (vs maintenance)	1.46	1.14–1.87	1.24	0.95–1.62
Commercial/Medicare (vs Medicaid)	1.28	0.92–1.84	1.28	0.88–1.88
Year of index rituximab > 2015 (vs 2011–2015)	1.26	1.01–1.57	1.22	0.96–1.54
Hospital admission without intensive care (vs no hospitalization)^a^	1.99	1.54–2.57	1.56	1.16–2.09
≥ 1 intensive care unit admission (vs no hospitalization)^a^	2.28	1.72–3.02	1.95	1.39–2.73
Serious infection^a^	1.53	1.06–2.18	0.89	0.59–1.34
Co-morbidity^b^	0.96	0.77–1.19	0.91	0.71–1.17
Prednisone 1–19 mg/day (vs none)^c^	2.86	2.00–4.06	2.63	1.83–3.77
Prednisone ≥ 20 mg/day (vs none)^c^	4.92	3.84–6.33	3.96	3.04–5.19
Azathioprine^a^	0.94	0.55–1.54	–	–
Methotrexate^a^	1.55	1.12–2.13	1.48	1.04–2.09
Cyclophosphamide^a^	1.02	0.53–1.81	–	–

^a^In the 6 months prior to the first rituximab

^b^At least one International Classification of Diseases diagnostic code in physician billing or hospitalization data for obstructive lung disease (asthma, bronchiectasis, chronic obstructive pulmonary disease), interstitial lung disease, diabetes, chronic kidney disease, or dialysis

^c^Dispensed in the month prior to the first rituximab

In the new user design group (with 6 months of continuous insurance enrollment prior to index RTX, *N* = 919), 281 (31%) were dispensed TMP-SMX at baseline. Multivariable analyses in this subgroup showed similar estimates to the primary analysis (Table 3).

**Table 3:** Multivariable logistic regression analysis of factors associated with TMP-SMX use, new user design^a^ (*N* = 919)

Characteristic at the time of first rituximab treatment	OR	95% CI
Age (years)	0.99	0.98–0.99
Female sex	0.57	0.42–0.77
Commercial/Medicare (vs Medicaid)	1.68	0.93–3.15
Year of index date (2011–2015 vs > 2015)	1.17	0.86–1.59
Rituximab induction (vs maintenance)	1.40	0.98–2.02
Hospital admission without intensive care (vs no hospitalization)^b^	2.59	1.78–3.78
≥ 1 intensive care unit admission (vs no hospitalization)^b^	2.93	1.91–4.50
Serious infection^b^	0.71	0.44–1.14
Co-morbidity^c^	0.88	0.64–1.21
Prednisone 1–19 mg/day (vs none)^d^	1.77	1.07–2.89
Prednisone ≥ 20 mg/day (vs none)^d^	2.27	1.58–3.29
Methotrexate^b^	1.32	0.83–2.07

^a^6 months of continuous insurance enrollment prior to rituximab

^b^In the 6 months prior to rituximab

^c^At least one International Classification of Diseases diagnostic code in physician billing or hospitalization data for obstructive lung disease (asthma, bronchiectasis, chronic obstructive pulmonary disease), interstitial lung disease, diabetes, chronic kidney disease, or dialysis

^d^Dispensed in the month prior to rituximab

### TMP-SMX use in the 6 months following rituximab

Expanding the definition of TMP-SMX use to include ≥ 28-day prescriptions any time in the 6 months following index RTX, we identified 609 TMP-SMX users (183 additional users compared to the primary analysis). Multivariable analysis in the entire cohort (*N* = 1877; 32% TMP-SMX users) and in the subgroup with 6 months of continuous insurance enrollment following RTX (*n* = 1308; 38% TMP-SMX users) showed similar results to the primary analyses (Additional file 1: Table S2).

### Sex-stratified analyses

To further characterize the negative association between female sex and TMP-SMX use, we stratified clinical characteristics according to sex (Additional file 1: Table S3). Females more frequently had Medicaid (14% vs 10%, difference 4%; 95% CI 2–7%), less frequently had a hospital admission (38% vs 45%, difference 6%; 95% CI 2–10%) and had fewer serious infections (6% vs 11%; difference 4%; 95% CI 2–7%) in the 6 months prior to RTX compared to males. In addition, chronic kidney disease (21% vs 26%, difference 5%; 95% CI 1–9%) and prednisone ≥ 20 mg/day in the prior month (31% vs 36%, difference 5%; 95% CI 1–9%) were less common in females compared to males. When multivariable logistic regression analyses were performed in males and females separately, younger age, rituximab induction (vs maintenance), and prior methotrexate use were significantly associated with TMP-SMX use in females, but we were unable to make definitive conclusions in males (Additional file 1: Table S4).

### TMP-SMX persistence

Among 389 TMP-SMX users with $\ge$ 1 new prescription following index RTX, the median persistence was 141 (Interquartile range 83, 248) days, with 163 (42%) continuing for 6 months or more. In univariable Cox proportional hazards regression analyses, both prednisone ≥ 20 mg/day in the month prior to RTX (HR 1.25; 95% CI 0.98–1.58) and hospitalization in the 6 months prior to RTX (HR 1.24; 95% CI 0.98–1.57) were potentially associated with TMP-SMX persistence (Additional file 1: Table S5).

### TMP-SMX prophylaxis alternatives

In the 6 months prior to receiving RTX, 38 (2%) had received at least one prescription for dapsone and 43 (2%) received atovaquone. Similarly, in the 6 months following RTX administration, 41 (2%) received a prescription for dapsone and 48 (3%) received atovaquone.

## Discussion

Rituximab is an important therapy for severe GPA, the most common form of AAV in North America [23, 29]. Recent practice guidelines conditionally recommended prophylaxis with TMP-SMX (or alternative) during RTX treatment in AAV (regardless of glucocorticoid dose), acknowledging limited data [2, 3, 21, 30], but little is known about prophylaxis patterns in the real world prior to these recommendations. Based on this large population sample, we estimate that 23–38% of RTX-treated patients with GPA are dispensed TMP-SMX prophylaxis at the time of RTX treatment, depending on the cohort and prophylaxis definitions used. Among TMP-SMX users, the median persistence was nearly 5 months, with 42% continuing prophylaxis for at least 6 months following index RTX.

Therapeutic trials in AAV often leave decisions on antibiotic prophylaxis to local practice [31–33] and rarely report the use of prophylaxis with the study results. In the RAVE trial (which compared RTX to cyclophosphamide for GPA and MPA induction) [34], where the majority of subjects received TMP-SMX prophylaxis (as intended in the protocol), the use of TMP-SMX was associated with reduced serious infections [20]. While studies have not assessed the effects of TMP-SMX prophylaxis during RTX maintenance alone, one retrospective study that included patients with repeated RTX infusions over time (mean cumulative RTX 4.75 g with 22 months follow-up) found that TMP-SMX was associated with reduced time to serious infection [18]. In this European tertiary care cohort, 38% of participants were taking TMP-SMX, which is slightly higher than our overall study estimate, but on par with the new user design subgroup (31%). In contrast, at an American tertiary care center, two-thirds of GPA patients (with various treatments) and 68% of RTX users overall (including other systemic rheumatic diseases) were prescribed some form of PJP prophylaxis [26]. However, this study captured intended TMP-SMX prescriptions as recorded in electronic health records, which may be higher than TMP-SMX actually dispensed to patients (as measured in our study). In our study, patients recently taking high-dose prednisone ($\ge$ 20 mg/day) were especially likely to receive TMP-SMX (39%), which likely reflects an increased perceived risk of PJP and/or other infections in this group. Furthermore, both prednisone use and prior hospitalization were associated with prophylaxis and potentially with TMP-SMX persistence. Higher acuity healthcare encounters and/or high disease activity might increase contact with tertiary care specialists, which could in turn provide more opportunities to prescribe and maintain prophylaxis.

Unexpectedly, females were less likely to be dispensed TMP-SMX. Prior studies did not find that antibiotic prophylaxis differed according to patient sex in AAV [19] or in other systemic diseases treated with immunosuppressants [26, 35] or RTX [27, 36]. In a Japanese prospective inception cohort, the female sex was protective against serious infections in AAV (adjusted HR 0.47 [95% CI 0.25,0.89]) [6], and we observed a small but significant difference in serious infections (in the 6 months prior to RTX) between females (6%) and males (11%) in our cohort. Conversely, the incidence of sulfa antibiotic allergy and antibiotic-associated adverse events might be higher in females [37, 38]. Thus, providers may prescribe TMP-SMX less to female patients due to perceived lower infection risk or concern for adverse events. Interestingly, in our sex-stratified analyses, younger age, RTX induction (vs maintenance), and prior methotrexate use were associated with TMP-SMX in females but not in males. Older females may be less likely to be dispensed TMP-SMX due to known allergies or intolerances identified earlier in life. However, we did not see a difference in the use of atovaquone or dapsone (which might indicate a known TMP-SMX allergy) according to sex. While our results should be interpreted with caution, especially as we lacked granular data to determine whether the omission of TMP-SMX prophylaxis was clinically justified, prior studies have observed sex disparities in the use of preventative treatments in other conditions such as diabetes [39] and peripheral artery disease [40]. The relationship between biological sex (and gender, which we were unable to evaluate) and AAV treatment and prophylaxis choices will require further study.

This large population-based cohort was similar in terms of demographics to other AAV cohorts in the USA [29, 41] and included subjects from different healthcare payors, which adds generalizability of our findings. Nevertheless, our study has limitations. GPA carries a high rate of hospitalization (> 40% of our cohort were hospitalized in the 6 months prior to RTX), but medications administered in the hospital are not captured in MarketScan. Thus, if RTX was only administered in the hospital, a patient may have been inadvertently excluded from the cohort. The lack of data on inpatient medications may have also led to incomplete ascertainment of prednisone exposure among hospitalized patients, potentially explaining the low measured prevalence of prednisone use in our cohort in the month prior to index RTX (34% taking $\ge$ 20 mg/day). Furthermore, the RTX dose was not available in the database, and our induction definition (≥ 2 RTX infusions within 30 days) may have misclassified maintenance RTX as induction if patients received 2 doses of 500 mg (rather than a single maintenance dose), or misclassified RTX induction as maintenance if only one of the infusions was administered as an outpatient (and therefore captured). However, sensitivity analysis with a new user design (which might enrich the population with induction recipients) found similar estimates. Our study is unique to differentiate RTX induction and maintenance therapy within administrative health data, which is an important consideration for future pharmacoepidemiologic studies in AAV. Although disease activity/severity and kidney function (which could influence TMP-SMX prescribing) are not directly measurable in the database, we included proxies of these measures in our analyses, including prior ICU admission, CYC use, CKD, and dialysis. We only measured TMP-SMX dispensed to the patient, which may underestimate providers’ intended prescriptions. Finally, while we did not differentiate prophylactic from therapeutic doses of TMP-SMX (i.e., double strength twice daily), we expect the minority of patients to be prescribed long-term therapeutic dose TMP-SMX in the last decade, as it is no longer a preferred disease-modifying agent [3].

## Conclusions

In conclusion, TMP-SMX was dispensed to less than one-third of US patients with GPA receiving RTX between 2011 and 2020. In our cohort, recent prednisone use and prior hospitalization were associated with TMP-SMX use, while females were less likely to receive prophylaxis. Antibiotic prophylaxis during RTX treatment in AAV is expected to increase following recent recommendations [2, 3, 21, 30]. Further work is needed to determine the association of TMP-SMX use with infectious outcomes in this population, in order to strengthen the evidence on optimal use of TMP-SMX during RTX treatment in AAV.

## Supplementary Information

**Additional file 1: Supplementary Table S1.** ICD-9 and ICD-10 inpatient primary diagnosis codes to define serious infection. **Supplementary Table S2.** Multivariable logistic regression analysis of factors associated with TMP-SMX use within 6 months of index rituximab. **Supplementary Table S3.** Sex-stratified cohort characteristics overall and according to TMP-SMX use. **Supplementary Table S4.** Multivariable logistic regression analysis of factors associated with TMP-SMX use, stratified by sex. **Supplementary Table S5.** Univariable Cox proportional hazards regression for factors associated with time to TMP-SMX discontinuation (*N* = 389^a^).

## Data Availability

The data underlying this article are available from Merative™ Marketscan®, but restrictions apply to the availability of these data, which were used under license for the current study, and so are not publicly available. Aggregate data will be shared upon request to the corresponding author with permission from Merative™ Marketscan®.

## Abbreviations

RTX: Rituximab
AAV: Anti-neutrophil cytoplasm antibody-associated vasculitis
GPA: Granulomatosis with polyangiitis
MPA: Microscopic polyangiitis
TMP-SMX: Trimethoprim-sulfamethoxazole
CYC: Cyclophosphamide
MTX: Methotrexate
ICU: Intensive care unit
PJP: Pneumocystis jirovecii pneumonia
US: United States
ICD: International Classification of Diseases (ICD)
CPT: Current Procedural Terminology
NDC: National Drug Code
