# Supplementary material for: Trimethoprim-sulfamethoxazole prophylaxis during treatment of granulomatosis with polyangiitis with rituximab in the United States of America: a retrospective cohort study
Source: Arthritis Res Ther. 2023 Jul 29;25:133. doi: 10.1186/s13075-023-03114-7 (PMC10386686; doi:10.1186/s13075-023-03114-7)
Supplement: Supplementary file 1 — Additional file 1: Supplementary Table S1. ICD-9 and ICD-10 inpatient primary diagnosis codes to define serious infection. Supplementary Table S2. Multivariable logistic regression analysis of factors associated with TMP-SMX use within 6 months of index rituximab. Supplementary Table S3. Sex-stratified cohort characteristics overall and according to TMP-SMX use. Supplementary Table S4. Multivariable logistic regression analysis of factors associated with TMP-SMX use, stratified by sex. Supplementary Table S5. Univariable Cox proportional hazards regression for factors associated with time to TMP-SMX discontinuation (N = 389a). [file 13075_2023_3114_MOESM1_ESM.docx]

Supplementary Table S1. ICD-9 and ICD-10 inpatient primary diagnosis codes to define serious infection

| Infection type | ICD-9 | ICD-10 |
| --- | --- | --- |
| Bacteremia/sepsis/septic shock | 036.2, 038.x, 790.7, 785.52, 995.91, 995.92 | A39.4, A40.9, A40.3, A41.89, A41.9, A41.4,  R78.81, R65.21. R65.20 |
| Head and neck/upper airway | 382.xx, 383.0x, 461.xx , 464.0x, 465.0x, 466.0x, 475, 478.22, 478.24, 380.1x | H66. H70.0x, J01.x, J04.0, J06.0, J06.9, J20.9, J36, J39.0, H60.x |
| Pulmonary | 481.x- 485.x, 486.x, 513.1, 484.3x, 484.5x,  415.12, 511.1, 510.9x 003.22 | J13, J18.x, J15.x, J14, J16.x, J17, J85.3, A37.91, A22.1, I26.90, J90, J86.9 |
| Central nervous system | 320.x, 036.0x, 003.21 | G00.0, A39.0, A02.21 |
|  | 324.xx | G06.x |
|  | 323.4x, 323.9x, 036.1x | G04.9x, G05.x, A39.81 |
| Soft tissue infection | 680.x - 682.x, 684.x, 035.x, 566.x, 686.8, 686.9, 785.4, 528.3, 728.0, 728.86, 376.01 | L02.x, L03.0x, L03.3x, A46, K61.x, L08.89, L08.9, I96, K12.2, M60.009, 728.86, H05.019, M72.6, A48 |
| Endocarditis | 421.0, 421.1, 421.9, 036.42, 098.84 | I33.0, I33.9, I39, A39.51, A54.83 |
| Gastrointestinal infection | 002.x - 005.x, 008.x, 009.x | A01.x, A02.x, A03.x, A05.x, A04.x, A09 |
| Hepatobiliary infection | 567.x, 572.0, 576.1, 569.5, 590.2, 614.4, 998.59 | K65.8, K67, K65.9, K75.0, K83.0, K63.0, K68.11 |
| Genitourinary infection | 595.xx, 599.0x, 098.12, 601.2x | N41.2, N30.01, N30.0x, N41.2 |
| Septic arthritis | 711.0x, 098.5x | M00.x A54.4x |
| Osteomyelitis | 730.0x, 730.1x, 730.2x, 730.8x, 003.24, | M86.x, M90.8xx, A02.24 |
| Other bacterial infections | 041.xx | B95.x |
| Other opportunistic infections | 039.x, 130.x | L08.1, A42.x, A43.9, B47.1, B58 |
| Pneumocystis | 136.3 | B59 |

Supplementary Table S2. Multivariable logistic regression analysis of factors associated with TMP-SMX use within 6 months of index rituximab

|  | Entire cohort  N=1877 | | Subgroup with 6 months continuous insurance enrolment following RTX N=1308 | |
| --- | --- | --- | --- | --- |
| Characteristic at the time of first rituximab treatment | OR | 95% CI | OR | 95% CI |
| Age (years) | 0.99 | 0.99-1.00 | 1.00 | 0.99-1.00 |
| Female sex | 0.69 | 0.56-0.85 | 0.64 | 0.50-0.81 |
| Commercial/Medicare (vs. Medicaid) | 1.52 | 1.08-2.16 | 1.11 | 0.73-1.70 |
| Year of index date >2015  (vs 2011-2015) | 1.32 | 1.07-1.63 | 1.05 | 0.82-1.34 |
| Rituximab induction (vs maintenance) | 1.32 | 1.04-1.69 | 1.64 | 1.24-2.16 |
| Hospital admission without intensive care (vs no hospitalization)^a^ | 1.42 | 1.08-1.85 | 1.61 | 1.18-2.20 |
| $\geq$1 intensive care unit admission  (vs no hospitalization)^a^ | 1.71 | 1.25-2.34 | 2.05 | 1.42-2.98 |
| Serious infection^a^ | 1.07 | 0.73-1.57 | 0.89 | 0.57-1.39 |
| Co-morbidity^b^ | 0.82 | 0.65-1.02 | 0.75 | 0.58-0.96 |
| Prednisone 1-19 mg/day  (vs none)^c^ | 2.39 | 1.73-3.29 | 1.54 | 1.08-2.20 |
| Prednisone $\geq$20 mg/day  (vs none)^c^ | 4.03 | 3.19-5.12 | 2.54 | 1.93-3.34 |
| Methotrexate^a^ | 1.32 | 0.95-1.83 | 1.11 | 0.78-1.57 |

^a^ in the 6 months prior to rituximab

^b^ At least one International Classification of Diseases diagnostic code in physician billing or hospitalization data for obstructive lung disease (asthma, bronchiectasis, chronic obstructive pulmonary disease), interstitial lung disease, diabetes, chronic kidney disease, or dialysis

^c^ in the month prior to rituximab

Supplementary Table S3. Sex-stratified cohort characteristics overall and according to TMP-SMX use

| Characteristic at time of first rituximab treatment | Female overall n=1008 | Female | | Male  overall  n=869 | Male | |
| --- | --- | --- | --- | --- | --- | --- |
|  |  | TMP-SMX  n=188 | No TMP-SMX  n=820 |  | TMP-SMX  n=238 | No TMP-SMX  n=631 |
| Age, mean (SD) | 50.8 (16.2) | 46.3 (15) | 51.9 (16) | 51.0 (15.4) | 50.1 (15) | 51.3 (16) |
| Insurance type, n (%) |  |  |  |  |  |  |
| Commercial | 728 (72) | 152 (81) | 576 (70) | 665 (77) | 193 (81) | 472 (75) |
| Medicare | 135 (14) | 11 (6) | 124 (15) | 118 (14) | 26 (11) | 92 (15) |
| Medicaid | 145 (14) | 25 (13) | 120 (15) | 86 (10) | 19 (8) | 67 (11) |
| Year of index date, n (%) |  |  |  |  |  |  |
| 2011-2015 | 486 (48) | 80 (43) | 406 (50) | 412 (47) | 105 (44) | 307 (49) |
| 2016-2019 | 522 (52) | 108 (57) | 414 (51) | 457 (53) | 133 (56) | 324 (51) |
| Rituximab treatment type, n (%)  Induction  Maintenance | 707 (70)  301 (30) | 150 (80)  38 (20) | 557 (68)  263 (32) | 607 (70)  262 (30) | 173 (73)  65 (27) | 434 (69)  197 (31) |
| Healthcare use, n (%)^a^ |  |  |  |  |  |  |
| $\geq$20 physician visits | 302 (30) | 72 (38) | 230 (28) | 275 (32) | 92 (39) | 183 (29) |
| Hospital admission | 386 (38) | 101 (54) | 285 (35) | 388 (45) | 135 (57) | 253 (40) |
| Intensive care unit admission | 162 (16) | 41 (22) | 121 (15) | 155 (18) | 61 (26) | 94 (15) |
| Serious infection | 65 (6) | 16 (9) | 49 (6) | 94 (11) | 32 (13) | 62 (10) |
| Disease features and/or co-morbidities, n (%) |  |  |  |  |  |  |
| Sinusitis | 255 (25) | 49 (26) | 206 (25) | 236 (27) | 67 (28) | 169 (27) |
| Obstructive lung disease | 214 (21) | 39 (21) | 175 (21) | 179 (21) | 57 (24) | 122 (19) |
| Interstitial lung disease | 54 (5) | 11 (6) | 43 (5) | 36 (4) | 8 (3) | 28 (4) |
| Glomerulonephritis | 109 (11) | 21 (11) | 88 (11) | 99 (11) | 25 (11) | 74 (12) |
| Chronic kidney disease | 211 (21) | 28 (15) | 183 (22) | 224 (26) | 63 (26) | 161 (26) |
| Dialysis | 84 (8) | 12 (6) | 72 (9) | 88 (10) | 28 (12) | 60 (10) |
| Diabetes | 141 (14) | 21 (11) | 120 (15) | 132 (15) | 35 (15) | 97 (15) |
| Medication use, n (%) |  |  |  |  |  |  |
| Prednisone 1-19 mg/day ^b^ | 114 (11) | 28 (15) | 86 (11) | 106 (12) | 32 (13) | 74 (12) |
| Prednisone $\geq$20 mg/day^b^ | 316 (31) | 106 (56) | 210 (25) | 314 (36) | 141 (59) | 173 (27) |
| Cyclophosphamide^a^ | 33 (3) | 6 (3) | 27 (3) | 28 (3) | 8 (3) | 20 (3) |
| Azathioprine^a^ | 50 (5) | 10 (5) | 40 (5) | 42 (5) | 10 (4) | 32 (5) |
| Methotrexate^a^ | 121 (12) | 34 (18) | 87 (11) | 81 (9) | 27 (11) | 54 (9) |
| Atovaquone^a^ | 20 (2) | 0 (0) | 20 (2) | 23 (3) | 4 (2) | 19 (3) |
| Dapsone^a^ | 24 (2) | 2 (1) | 22 (3) | 14 (2) | 1 (0) | 13 (2) |

^a^ in the 6 months prior to RTX

^b^ in the month prior to RTX

Supplementary Table S4. Multivariable logistic regression analysis of factors associated with TMP-SMX use, stratified by sex

| Characteristic at the time of first rituximab treatment | Females N=1008 | | Males N=869 | |
| --- | --- | --- | --- | --- |
|  | OR | 95% CI | OR | 95% CI |
| Age (years) | 0.98 | 0.96-0.99 | 1.00 | 0.99-1.01 |
| Commercial/Medicare (vs. Medicaid) | 1.31 | 0.79-2.25 | 1.29 | 0.74-2.34 |
| Year of index date >2015  (vs 2011-2015) | 1.29 | 0.91-1.83 | 1.16 | 0.84-1.61 |
| Rituximab induction (vs maintenance) | 1.61 | 1.06-2.47 | 1.01 | 0.71 to 1.44 |
| Hospital admission without intensive care (vs no hospitalization)^a^ | 1.89 | 1.22-2.92 | 1.35 | 0.91 - 2.00 |
| $\geq$1 intensive care unit admission  (vs no hospitalization)^a^ | 1.96 | 1.18 - 3.22 | 1.91 | 1.20 - 3.04 |
| Serious infection^a^ | 0.95 | 0.48-1.84 | 0.87 | 0.51 - 1.47 |
| Co-morbidity^b^ | 0.90 | 0.62-1.29 | 0.91 | 0.65 -1.28 |
| Prednisone 1-19 mg/day  (vs none)^c^ | 2.88 | 1.68 - 4.86 | 2.44 | 1.47 - 4.01 |
| Prednisone $\geq$20 mg/day  (vs none)^c^ | 3.81 | 2.57 - 5.70 | 4.19 | 2.91 - 6.07 |
| Methotrexate^a^ | 1.80 | 1.11 - 2.88 | 1.14 | 0.67-1.91 |

^a^ In the 6 months prior to rituximab

^b^ At least one International Classification of Diseases diagnostic code in physician billing or hospitalization data for obstructive lung disease (asthma, bronchiectasis, chronic obstructive pulmonary disease), interstitial lung disease, diabetes, chronic kidney disease, or dialysis

^c^ In the month prior to rituximab

Supplementary Table S5. Univariable Cox proportional hazards regression for factors associated with time to TMP-SMX discontinuation (N=389^a^)

| Characteristic at the time of first rituximab treatment | HR | 95% CI |
| --- | --- | --- |
| Age (years) | 1.00 | 0.99 - 1.01 |
| Female sex | 0.93 | 0.73 - 1.18 |
| Commercial/Medicare (vs. Medicaid) | 1.23 | 0.85 - 1.77 |
| Rituximab induction (vs maintenance) | 1.00 | 0.75 - 1.33 |
| Prednisone $\geq$20 mg/day^a^ | 1.25 | 0.98 - 1.58 |
| Hospitalized^b^ | 1.24 | 0.98 - 1.57 |
| Co-morbidity^c^ | 1.02 | 0.81 - 1.30 |

^a^ Excludes n=37 subjects who were not dispensed a new TMP-SMX prescription after rituximab index date

^b^ in month prior to rituximab

^c^ in 6 months prior to rituximab

^d^ at least one International Classification of Diseases diagnostic code in physician billing or hospitalization data for obstructive lung disease (asthma, bronchiectasis, chronic obstructive pulmonary disease), interstitial lung disease, diabetes, chronic kidney disease, or dialysis

Abbreviations: TMP-SMX, trimethoprim sulfamethoxazole
